# A Systematic Review and Meta-Analysis of the Outcomes of Laparoscopic Cholecystectomy Compared to the Open Procedure in Patients with Gallbladder Disease

**DOI:** 10.1055/s-0043-1777710

**Published:** 2024-02-01

**Authors:** Debajit Kumar Roy, Rahaman Sheikh

**Affiliations:** 1 Department of Surgery, R G Kar Medical College & Hospital, West Bengal University of Health Sciences, RG Kar Road, Kolkata, West Bengal, India; 2 Department of Anaesthesia, NRS Medical College, Kolkata, West Bengal, India

**Keywords:** open cholecystectomy, laparoscopic cholecystectomy, gallbladder disease, cholecystectomy

## Abstract

**Background** Conflicting evidence regarding the laparoscopic versus open cholecystectomy outcomes in scientific literature impacts the medical decision-making for patients with gallbladder disease. This study aimed to compare a range of primary and secondary outcomes between patients receiving laparoscopic cholecystectomy and those with open intervention.

**Methods** Articles published from 1993 to 2023 were explored by utilizing advanced filters of PubMed Central/Medline, Web of Science, CINAHL, JSTOR, Cochrane Library, Scopus, and EBSCO. The gallbladder disease was determined by the presence of one or more of the following conditions: 1) Gangrenous cholecystitis, 2) acute cholecystitis, 3) chronic gallbladder diseases, and 4) cholelithiasis. The primary end-point was mortality, while the secondary outcome included (1) bile leakage, 2) common bile duct injury, 3) gangrene, 4) hospital stay (days), 5) major complications, 6) median hospital stay (days), (7) pneumonia, 8) sick leaves (days), and 9) wound infection.

**Results** Statistically significant reductions were observed in mortality (odds ratio [OR]: 0.30, 95% confidence interval [CI]: 0.30, 0.45, *p* < 0.00001), mean hospital stay duration (mean difference: –2.68, 95% CI: –3.66, –1.70, *p* < 0.00001), major complications (OR: 0.35, 95% CI: 0.19, 0.64, *p* = 0.0005), post/intraoperative wound infection (OR: 0.29, 95% CI: 0.16, 0.51, *p* < 0.0001), and sick leaves (OR: 0.34, 95% CI: 0.14, 0.80, *p* = 0.01) in patients who underwent laparoscopic cholecystectomy compared with those with the open intervention. No statistically significant differences were recorded between the study groups for bile leakage, common bile duct injury, gangrene, median hospital stay days, and pneumonia ( *p* > 0.05).

**Conclusions** The pooled outcomes favored the use of laparoscopic cholecystectomy over the open procedure in patients with gallbladder disease. The consolidated findings indicate the higher impact of laparoscopic cholecystectomy in improving patient outcomes, including safety episodes, compared with open cholecystectomy.

## Introduction

Gallbladder disease progresses with gallbladder blockage, infection, or inflammation. The clinical manifestations of gallbladder disease include cholecystitis, gallstones, empyema/abscess, gangrene, sclerosing cholangitis, acalculous condition, and abnormal tissue growth. 1 The impaired gallbladder emptying or cystic duct occlusion results in the development of gallbladder inflammation/acute cholecystitis and gallstones/cholelithiasis (with an incidence rate of 95%). 2 The solidification of bile juice inside the gallbladder results in cholelithiasis, which impacts 9% of females and 6% of males in the United States; 1 to 2% of asymptomatic patients with cholelithiasis have a risk of developing clinical complications. 3 Cystic duct obstruction and bile stasis trigger the development of gallbladder empyema in 5 to 15% of patients with acute cholecystitis. 4 Vascular insufficiency, ischemia, perforation, and necrosis of the gallbladder progressively trigger gangrenous cholecystitis in patients with diabetes, cardiovascular disease, leukocytosis, delayed surgery, advanced age, and male gender. In addition, a 15 to 50% mortality rate in gallbladder disease corresponds to gangrenous cholecystitis. 5 Primary sclerosing cholangitis is characterized by biliary strictures, fibrosis, and inflammation. A high annual incidence (0–1.3/10 ^5^ individuals/year) of this condition is reported in 65 to 70% of males versus females 6 ; less than 10% of patients with acute gallbladder inflammation develop gallbladder infection without calculous, which is known as acalculous cholecystitis. 7 Benign gallbladder conditions include diffuse wall thickening and intraluminal lesions whose abnormal growth may result in carcinomatous conditions. 8

Tokyo guidelines (2018) guide the diagnostic assessment and management of acute cholangitis. 9 In addition, evidence emphasizes asymptomatic cholelithiasis treatment with conservative approaches, while surgical interventions are recommended for porcelain/calcified gallbladder, including more than 1 cm polyps and more than 3cm gallstones. In general, open surgery is preferred over the laparoscopic approach only in case of choledocholithiasis suspicion, high bleeding, or problematic anatomical identification. 10 Laparoscopic surgery outweighs the open approach due to a lower incidence of infections and bile duct injuries. The surgical management of gallstones/cholecystitis is undertaken via cholecystectomy with/without antibiotic prophylaxis. Laparoscopic cholecystectomy is recommended for the early management of acute cholecystitis within a day of hospital admission. 11 The four-trocar procedure is the method of choice for laparoscopic cholecystectomy, which should not be delayed above 3 days of diagnostic confirmation, to avoid clinical complications. Conflicting evidence regarding the laparoscopic versus open cholecystectomy outcomes in scientific literature impacts the medical decision-making for patients with gallbladder disease. A meta-analysis by Keus et al indicated faster recovery and reduced hospital stay following the laparoscopic cholecystectomy compared with the open approach. 12 However, the authors did not find any significant difference between the two approaches for operative time, clinical complications, and mortality. Alternatively, the meta-analysis by Antoniou et al reveals a statistically significant reduction in cardiorespiratory complications, morbidity, and mortality in elderly patients undergoing laparoscopic cholecystectomy compared with open surgery. 13 A systematic review and meta-analysis by Coccolini et al indicates a marked reduction in wound infection incidence, pneumonia rates, hospital stay, morbidity, and mortality following laparoscopic cholecystectomy compared with the open intervention. 14 However, this study does not delineate differences in bile leakage and severe hemorrhage incidence rates following both interventions.

Additionally, clinical guidelines do not recommend open/laparoscopic cholecystectomy in gallbladder disease without ruling out the risk/incidence of gallbladder cancer. 15 The routine indications of laparoscopic cholecystectomy include chronic/acute cholecystitis, symptomatic cholelithiasis, biliary dyskinesia, acalculous cholecystitis, gallstone pancreatitis, and gallbladder polyps/masses. The contraindications include metastasis, unmanageable coagulopathy, and intolerance to general anesthesia/pneumoperitoneum. The conversion of laparoscopic cholecystectomy to open surgery depends on the risk of clinical complications, comorbidities, and other potential factors including elevated amylase, high blood glucose/leucocyte levels, morbidity incidence, past abdominal surgery, obesity, elevated white blood count, and male gender. 16 17 The risk assessment for these factors is necessary to select an appropriate cholecystectomy procedure for gallbladder disease. The contemporary literature does not provide standard conventions to differentiate the need for laparoscopic versus open cholecystectomy for patients with gallbladder disease. The lack of recent meta-analyses and corresponding latest evidence in this regard makes medical decision-making increasingly difficult for patients with cholecystitis/cholelithiasis. Accordingly, this systematic review and meta-analysis aimed to consolidate findings and compare the outcomes of laparoscopic cholecystectomy with open surgery in patients with gallbladder disease. To the best of our knowledge, this is the most recent meta-analysis to date, comparing a range of primary and secondary outcomes between patients receiving laparoscopic cholecystectomy and those with the open procedure.

## Materials and Methods

### Literature Search and Data Collection Approaches

Two authors independently explored articles of interest across databases including PubMed Central/Medline, EMBASE, Web of Science, CINAHL, JSTOR, Cochrane Library, Scopus, and EBSCO. Articles published from 1993 to 2023 were explored by utilizing advanced filters of the corresponding databases. The search terms/Medical Subject Headings included “cholecystectomy,” “open,” “laparoscopic,” “gallbladder disease,” “surgery,” “cholelithiasis,” “cholecystitis,” “gallstones,” “cholangitis,” and “inflammation.” Boolean operators were used to formulate various search term combinations targeting the studies of interest. Title/abstract-based searches were followed by journal-based searches for extracting the relevant articles. In addition, citations/references from relevant articles were manually researched to identify studies with the latest information matching the aim/objectives of this study. Full-text articles were eventually explored to reduce the risk of missing data/outcomes.

### Inclusion and Exclusion Parameters

This systematic review and meta-analysis included articles based on prospective nonrandomized, retrospective, and prospective randomized trials. The included studies examined open and laparoscopic cholecystectomies and their outcomes in adult/elderly patients who were diagnosed with any of the following conditions: 1) gangrenous cholecystitis, 2) acute cholecystitis, 3) chronic gallbladder diseases, and 4) cholelithiasis. These diagnoses were affirmed following the clinical parameters and standardized protocols of the individual clinical settings. This study did not include articles based on evidence-based reviews, systematic reviews, literature reviews, meta-analyses, editorials, and letters to the editor/correspondences. In addition, no randomized controlled trial was known to compare laparoscopic and open cholecystectomy till the date of screening, which was performed on April 1, 2023.

### Primary and Secondary Outcomes

The primary end-point was mortality, while the secondary outcome variables included the following: 1) bile leakage, 2) common bile duct injury, 3) gangrene, 4) hospital stay (days), 5) major complications, 6) median hospital stay (days), 7) pneumonia, 8) sick leaves (days), and 9) wound infection. The authors did not include open cholecystectomy conversion rates due to inconsistencies in the available data and a lack of updated recent data for this parameter.

### Risk of Bias Assessment

Two authors collaborated to construct risk of bias (ROB) graphs and summaries using ROBINS-I and ROB-2 Cochrane tools. 18 19 Thirteen prospective/retrospective nonrandomized studies were assessed for ROB via the ROBINS-1 tool, while seven randomized trials were investigated via the ROB-2 measure. The ROB domains assessed via the ROBINS-I tool included (1) confounding-related bias, 2) bias based on participant selection, 3) bias in intervention classification, 4) bias arising from deviations from the desired approaches, 5) bias in the context of missing data, 6) bias in outcome assessment, and 7) bias in reporting of outcomes. The overall bias was categorized into serious, moderate, low, and no information. The ROB via ROB2 measure was categorized into 1) bias due to randomization, 2) bias emanating from diversions from the intended strategies, 3) bias correlating with the missing results, 4) outcome measurement bias, and 5) outcome selection bias. The overall bias was categorized into some concerns, low bias, and no information.

### Statistical Analysis

The MS Excel spreadsheet was utilized for collecting the study data. 20 Double data entry checks by two independent authors nullified the risk and incidence of data collection errors. Cochrane's Review Manager (RevMan-5.4) tool was used for the statistical analysis of the collected data. 21 Odds ratios (ORs) were calculated within their 95% confidence intervals (CIs) for most of the outcome variables 22 ; OR less than 1 indicated a reduced incidence of the outcome of interest in patients with laparoscopic cholecystectomy versus those with open cholecystectomy. Alternatively, OR more than 1 indicated the opposite outcome. The assessment of mean differences (MDs) between the laparoscopic and open surgeries determined variations in hospital stay duration between both study groups. Weights obtained from each study indicated the impact of the corresponding outcome variable in patients with laparoscopic versus open cholecystectomy. The overall statistical analysis was guided by the Mantel–Haenszel random-effects approach. 23 For each end-point, the chi-squared, tau-squared, and I-squared statistics determined the heterogeneity differences between the included studies. 24 The minimal/low, moderate, and high heterogeneity levels were indicated by I-squared ranges of 0 to 50%, 51 to 80%, and 81 to 100%, respectively. Forest plots demonstrating the selected end-points facilitated their visual comparisons between the study groups.

## Results

### Screening of Articles

The articles of interest were screened across PubMed Central/Medline, Web of Science, CINAHL, JSTOR, SCOPUS, and Google Scholar. Citations from the included articles were manually screened further to avoid the risk of missing entries. Seven hundred and twenty-eight studies were explored via database searching, and 11 additional articles were identified via other sources. In total, 739 records were filtered after removing duplicates. Of them, 719 were discarded based on the inclusion/exclusion parameters, and 29 full-text articles were screened for eligibility. After removing five full-text articles, 24 studies were finalized for systematic review and meta-analysis ( Supplementary Fig. S1 [online only]), guided by the PRISMA (preferred reporting items for systematic reviews and meta-analyses) statement. 25

### Baseline Assessment and Systematic Review

Table 1 reports baseline characteristics and clinical history of participants in the included studies, while Table 2 summarizes the systematic review findings, categorized into author/study name, publication year, study design, sample size, end-points, interventions, and outcomes.

**Table 1:** Baseline characteristics

Characteristics	Study																																															
	Araujo-Texeira et al 1999 26		Feldman et al. 1994 27		Glavić et al 2001 28		Johansson et al 2005 29		Karim and Kadyal 2015 30		Kiviluoto et al 1998 31		Krähenbühl and Büchler 1996 32		Maxwell et al 1998 33		Pessaux et al 2001 34		Tucker et al 2011 35		Unger et al 1993 36		Catena et al /ACTIVE Study 37		Eldar et al 1997 38		Lujan et al 1998 39		Boo et al 2007 40		Chau et al 2002 41		Singh et al 2017 42		Zacks et al 2002 43		Massie et al 1993 46		Pandey et al 2019 48		Wu et al 2023 47		Agarwal et al 2023 44		Baruah et al 2022 49		Khalid et al 2023 45	
**LC**	OC	LC	OC	LC	OC	LC	OC	LC	OC	LC	OC	LC	OC	LC	OC	LC	OC	LC	LC	OC	LC	LC	OC	OC	LC	OC	LC	OC	LC	OC	LC	OC	LC	OC	LC	OC	LC	OC	LC	LC	OC	LC	OC	LC	OC	LC	OC
Patients ( *n* )	100	100	2269	545	349	35	35	50	50	32	31	20	51	5034	13466	50	89	10137	1789	100	100		72	72	2269	545	349	35	35	50	50	32	31	20	51	5034	13466	50	89	10137	44	119	31	29	200	200	40	40
Mean age (SD)/range (years)	62 (±15)	56 (±14)	≥65	–	–	–	53 (23–84)	56 (31–80)	–	–	61·4 (13·6)/28–82	58·9 (15·9)/ 25–88	–	–	83.9	84.0	81.9	81.9	–	62 (±15)	56 (±14)	≥65	–	–	–	–	–	53 (23–84)	56 (31–80)	–	–	61·4 (13·6)/28–82	58·9 (15·9)/ 25–88	–	–	83.9	84.0	81.9	81.9	–	66.0 ± 10.3	63.0 ± 9.3	–	–	36.21 ± 9.00	37.61 ± 8.75	40.025 ± 8.12	42.52 ± 8.76
Gender (F/M) ( *n* , %)	–	–	–	–	–	19:16	–	–	–	–	–	–	–	–	67%	62%	30	51	62.9%/37.1%	–	–	–	–	–	–	–	19:16	–	–	–	–	–	–	–	–	67%	62%	30	51	62.9%/37.1%	19/25	54/65	25/6	20/9	132/68	139/61	–	–
M:F sex ratio	–	–	–	–	–	–	19:16	16:19	–	–	–	–	–	–	–	–	–	–	–	–	–	–	–	–	–	–	–	19:16	16:19	–	–	–	–	–	–	–	–	–	–	–	–	–	–	–	–	–	–	–
Mean body weight (SD) (kg)	–	–	–	–	–	–	–	–	–	–	–	–	–	–	–	–	–	–	–	–	–	–	–	–	–	–	–	–	–	–	–	–	–	–	–	–	–	–	–	–	–	–	–	–	60.90 ± 11.21	69.12 ± 14.25	–	–
BMI (SD)/range (kg/m ^2^ ) ( *n* )	–	–	–	–	–	–	–	–	–	–	26·9 (0·8)	27·7 (0·9)	–	–	–	–	–	–	31 (27–36)	–	–	–	–	–	–	–	–	–	–	–	–	26·9 (0·8)	27·7 (0·9)	–	–	–	–	–	–	31 (27–36)	24.4 ± 3.5	23.8 ± 3.4	–	–	–	–	–	–
Comorbidity ( *n* , %)	–	–	–	–	–	72 (36–168)	–	–	–	–	–	–	–	–	–	–	–	–	–	–	–	–	–	–	–	–	72 (36–168)	–	–	–	–	–	–	–	–	–	–	–	–	–	–	–	–	–	–	–	–	–
Symptom duration (mean value)/range	–	–	–	––	–	–	72 (36–168)	72 (12–150)	–	–	3.5	4.8	–	–	–	–	–	–	–	–	–	–	–	–	–	–	–	72 (36–168)	72 (12–150)	–	–	3.5	4.8	–	–	–	–	–	–	–	–	–	–	–	–	–	–	–
Past surgery ( *n* , %)	–	–	–	–	–	–	–	–	–	–	–	–	–	–	–	–	–	–	–	–	–	–	–	–	–	–	–	–	–	–	–	–	–	–	–	–	–	–	–	–	–	–	–	–	–	–	–	–
ASA PSS class I–II ( *n* , %)	76%	84%	–	–	–	–	–	–	–	–	17	20	–	–	–	–	29 (58)	53 (59.6)	48.1%	76%	84%	–	–	–	–	–	–	–	–	–	–	17	20	–	–	–	–	29 (58)	53 (59.6)	48.1%	–	–	–	–	–	–	–	–
ASA PS class III––IV ( *n* , %)	24%	16%	–	–	–	–	–	–	–	–	15	11	–	–	–	–	2 (4)	2 (2.2)	3.1%	24%	16%	–	–	–	–	–	–	–	–	–	–	15	11	–	–	–	–	2 (4)	2 (2.2)	3.1%	–	–	–	–	–	–	–	–
Patients with prior intraabdominal surgery	–	–	–	–	–	–	–	–	–	–	9	8	–	–	–	–	–	–	–	–	–	–	–	–	–	–	–	–	–	–	–	9	8	–	–	–	–	–	–	–	–	–	–	–	–	–	–	–
Preoperative diffuse peritonitis	–	–	–	–	–	–	–	–	–	–	1	1	–	–	–	–	–	–	–	–	–	–	–	–	–	–	–	–	–	–	–	1	1	–	–	–	–	–	–	–	–	–	–	–	–	–	–	–
Preoperative gall bladder thickening	–	–	–	–	–	–	–	–	–	–	22	24	–	–	–	–	–	–	–	–	–	–	–	–	–	–	–	–	–	–	–	22	24	–	–	–	–	–	–	–	–	–	–	–	–	–	–	–
Mean (SD) CRP/range, mg/L	–	–	–	–	–	–	140 (23–290)	189 (6–340)	–	–	136·2 (88·6)	124·7 (83·1)	–	–	–	–	–	–	–	–	–	–	–	–	–	–	–	140 (23–290)	189 (6–340)	–	–	136·2 (88·6)	124·7 (83·1)	–	–	–	–	–	–	–	–	–	–	–	–	–	–	–
Mean (SD) leucocyte count, 10 ^9^ /L	–	–	–	–	–	–	–	–	–	–	9·03 (3·58)	11·3 (8·31)	–	–	–	–	–	–	–	–	–	–	–	–	–	–	–	–	–	–	–	9·03 (3·58)	11·3 (8·31)	–	–	–	–	–	–	–	–	–	–	–	–	–	–	–
Temperature (°C)	–	–	–	–	–	–	38·2 (36·3–39·0)	38·3 (36·5–39·7)	–	–	–	–	–	–	–	–	–	–	–	–	–	–	–	–	–	–	–	38·2 (36·3–39·0)	38·3 (36·5–39·7)	–	–	–	–	–	–	–	–	–	–	–	–	–	–	–	–	–	–	–
Preoperative fever ( *n* , %)	39%	31%	–	–	–	–	–	–	–	–	–	–	–	–	–	–	–	–	–	39%	31%	–	–	–	–	–	–	–	–	–	–	–	–	–	–	–	–	–	–	–	4	5	–	–	–	–	–	–
Preoperative gallbladder mass ( *n* , %)	–	–	–	–	–	–	–	–	–	–	–	–	–	–	–	–	–	–	–	–	–	–	–	–	–	–	–	–	–	–	–	–	–	–	–	–	–	–	–	–	–	–	–	–	–	–	–	–
Preoperative leukocytosis (n, %)	–	–	–	–	–	–	–	–	–	–	–	–	–	–	–	–	–	–	–	–	–	–	–	–	–	–	–	–	–	–	–	–	–	–	–	–	–	–	–	–	–	–	–	–	–	–	–	–
Preoperative deranged liver function ( *n* , %)	–	–	–	–	–	–	–	–	–	–	–	–	–	–	–	–	–	–	–	–	–	–	–	–	–	–	–	–	–	–	–	–	–	–	–	–	–	–	–	–	–	–	–	–	–	–	–	–
Preoperative ERCP ( *n* , %)	–	–	–	–	–	–	–	–	–	–	–	–	–	–	–	–	–	–	–	–	–	–	–	–	–	–	–	–	–	–	–	–	–	–	–	–	–	–	–	–	–	–	–	–	–	–	–	–
Preoperative concurrent common bile duct stone ( *n* , %)	–	–	–	–	–	–	–	–	–	–	–	–	–	–	–	–	–	–	–	–	–	–	–	–	–	–	–	–	–	–	–	–	–	–	–	–	–	–	–	–	–	–	–	–	–	–	–	–

Abbreviation: ASA-PSS, American Society of Anesthesiologists physical status score; BMI, body mass index; ERCP, endoscopic retrograde cholangiopancreatography; LC, laparoscopic cholecystectomy; OC, open cholecystectomy; SD, standard deviation.

**Table 2:** Systematic review

Authors/Study	Year	Study type/design	Sample size	Endpoints	Intervention(s)	Outcome(s)
Araujo-Texeira et al 26	1999	Prospective-nonrandomized	*N* = 200 [LC ( *N* = 100), OC ( *N* = 100)]	• Postoperative mortality • Postoperative morbidity • Hospital stay duration • OC conversion rate • Other clinical complications	This study compared LC and OC outcomes in patients with acute cholecystitis	While no death was reported in patients who received LC, a 2% mortality incidence was reported in the OC group Patients receiving LS had a comparatively shorter hospital stay (5 ± 3 vs. 12 ± 10, *p* = 0.00005) and lower morbidity rate (10% vs. 32%, *p* = 0.002) compared with those with LC
Feldman et al 27	1994	Prospective, multicenter, and nonrandomized trial	*N* = 2269 [LC ( *N* = 691 + 817), OC ( *N* = 761)]	• Mortality rates	This study compared mortality rates following LC and OC in patients (age ≥65 years) with chronic gallbladder diseases	Patients who underwent LC had a mortality rate range of 0.3–0.6% compared with 1.4% in patients with OC; the mortality rate further declined after LC in patients of age group 70–79 years ( *p* = 0.01)
Glavić et al 28	2001	Retrospective study	*N* = 209 [LC ( *N* = 94), OC ( *N* = 115)]	• Sick leaves • Mean operating durations • Wound infection • Biliary complications • Hospital cost	This study compared the cost-effectiveness and clinical outcomes of LC versus OC among patients with acute cholecystitis	Patients who underwent LC had a higher mean operative duration compared with those with OC (115 vs 89 minutes) No deaths versus 2 deaths within a month of surgical treatment were reported in patients with LC versus OC Two patients developed massive/prolonged biliary secretions after surgery in both LC and OC groups, respectively Wound infection was reported in 2.13% of patients who underwent LC versus 8.7% with OC Patients with OC had a significantly higher rehabilitation and sick leave cost compared with those with LC (USD1199 vs. USD486)
Johansson et al 29	2005	Prospective randomized trial	*N* = 70 [LC ( *N* = 35), OC ( *N* = 35)]	Perioperative, postoperative, and cost-related outcomes • Operative time • Conversion to OC • Perioperative bleeding • Clinical complications/morbidity • Postoperative hospital stay duration • Postoperative sick leaves • Pain score	This study compared the outcomes between patients who underwent OC and LC for acute cholecystitis	Patients who received OC had a statistically significant reduction in operative time compared with those with LC (80 vs. 90 minutes, *p* = 0.04) Patients in the LC group had a statistically significant decline in the postoperative hospital stay duration compared with those in the OC group ( *p* = 0.011) No differences regarding direct treatment expenditure were observed between both treatment arms
Karim and Kadyal 30	2015	Prospective randomized study	*N* = 100 [LC ( *N* = 50), OC ( *N* = 50)]	• Postoperative analgesia • Operative time • Postoperative hospital stay duration • Morbidity Mortality	This study compared the outcomes of LC and OC in patients with cholelithiasis	Open cholecystectomy was associated with a mean operative duration of 70 minutes versus 103.98 minutes with LC ( *p* < 0.001) Open cholecystectomy required a higher supply of injectable analgesics compared with LC (mean of 3.36 vs. 1.5 days)
Kiviluoto et al 31	1998	Prospective randomized trial	*N* = 63 [LC ( *N* = 32), OC ( *N* = 31)]	• Hospital morbidity/mortality • Hospital stay duration • Sick leave duration	This study compared the efficacy and clinical complications of LC versus OC in patients with gangrenous/acute cholecystitis	Empyema/gangrene was observed in 13 subjects in the LC group and 13 in the OC group; peritonitis derived from gallbladder perforation was observed in 1 patient in both LC and OC groups, respectively Open cholecystectomy conversion after an LC attempt was reported in 16% of patients; patients in the OC group had a higher incidence of postoperative complications compared with those who received LC ( *p* = 0·0048) Patients receiving LC had a 3% incidence rate of minor clinical manifestations/complexities versus 19% in those with OC Both mean sick leave (13.9 vs. 30.1 days, 95% CI: 10.9–21.7) and postoperative hospital stay (median of 4 days vs. 6 days; *p* = 0·0063) durations were significantly lower in patients who received LC compared with those with OC
Krähenbühl and Büchler 32	1996	Retrospective-with-historical-control-group	*N* = 71 [LC ( *N* = 20), OC ( *N* = 51)]	• Mortality • Morbidity/major complications • Hospital days • CBD injury rate • Bile leakage	This study compared the outcomes of OC and LC in patients with acute cholecystitis	The findings indicated the feasibility and safety of LC versus OC in the acute cholecystitis setting No statistically significant differences in CBD injury and mortality were observed in both patient groups
Maxwell et al 33	1998	Multicenter prospective study	*N* = 18500 [LC ( *N* = 5034), OC ( *N* = 13466)]	• Mortality Hospital stay duration	This study compared the outcomes of high-performance LC with OC in patients (age ≥80 years)	Patients with LC-guided cholecystectomy had a 1.8% lower mortality rate than those with OC; in addition, the death rate declined over time and was reduced in women than men
Pessaux et al 34	2001	Prospective nonrandomized study	N = 139 [LC ( *N* = 50), OC ( *N* = 89)]	• Operative duration • Postoperative hospital stay duration • Inpatient rehabilitation • Postoperative morbidity • OC conversion rate	This study compared the efficacy and feasibility of LC and OC in patients (age >75 years) who received treatment for acute cholecystitis	Patients who underwent OC had significantly increased inpatient rehabilitation (42 vs 15 patients), postoperative hospital stay duration (12.7 vs 7.7 days), and operative time (149.7 vs 103.3 minutes) compared with those who received LC for acute cholecystectomy Both study groups did not differ in terms of postoperative morbidity incidence; the OC group witnessed 4 deaths compared with 0 in the LC group
Tucker et al 35	2011	Prospective study	*N* = 11926 [LC ( *N* = 10137), OC ( *N* = 1789)]	• Hospital stay duration • Thirty-day complications • Mortality	This study investigated the safety outcomes of LC and compared them with OC in elderly versus nonelderly patients	Elderly patients who underwent OC had a higher mortality rate and longer hospital stay duration compared with those with LC ( *p* < 0.05); LC utilization was lower in elderly than non-elderly patients despite its reduced postoperative complications
Unger et al 36	1993	Retrospective study	*N* = 200 [LC ( *N* = 50), OC ( *N* = 50)]	• Mortality • CBD injury • Hospital stay duration	This study compared outcomes, cost, and clinical complications associated with LC versus OC in patients with acute cholecystitis	In comparison to OC-guided cholecystectomy, patients who underwent LC-guided cholecystectomy had early resumption of normal activities, reduced hospital stay duration, and comparable morbidity
Catena et al/ACTIVE Study 37	2013	Prospective randomized controlled multicenter trial	*N* = 144 [LC ( *N* = 72), OC ( *N* = 72)]	• OC conversion rate • Clinical complications/morbidity • Mortality • Hospital stay duration Wound infection	This study compared aesthetic results and postoperative outcomes of LC versus OC in the acute cholecystitis setting	In the LC group, OC conversion was required in 9.7% of patients; no bile duct lesions/deaths were reported in patients with LC or OC No statistically significant differences were reported for postoperative hospital stay and complication rates between the study groups
Eldar et al 38	1997	Prospective study	*N* = 243 [LC ( *N* = 146), OC ( *N* = 97)]	• Major complications • Pneumonia • Wound infection • Bile leakage • Median hospital stay duration	This study compared safety outcomes between LC and OC in patients with acute cholecystitis	Thirty safety outcomes were observed in 26% of patients who underwent cholecystectomy via OC compared with 16.5% of those with LC who experienced 25 complications
Lujan et al 39	1998	Prospective nonrandomized trial	*N* = 224 [LC ( *N* = 114), OC ( *N* = 110)]	• Operating time • OC conversion rate in the LC group • Clinical complications • Hospital stay duration	This study compared cholecystectomy outcomes between patients who were treated with LC and OC for acute cholecystitis	Patients with LC had 88 minutes of mean operative time compared with 77 minutes in those with OC ( *p* < 0.001) Clinical complications between the OC and LC groups were comparable (23% vs 14%, *p* = 0.06) Patients receiving OC had a higher incidence of mild complications and similar rates of severe/moderate complications compared with the LC group ( *p* < 0.02) Patients who received LC had a lower hospital stay duration compared with those with OC (3.3 vs 8.1 days, *p* < 0.001)
Boo et al 40	2007	Prospective randomized trial	*N* = 33 [LC ( *N* = 18), OC ( *N* = 15)]	• Postoperative morbidity • Hospital stay duration • Operation duration	This study compared immune responses and systemic inflammation in patients treated with OC/LC for acute cholecystitis	Patients with OC had significantly higher hospitalization compared with those with LC (6.3 ± 2.7 versus 3.7 ± 1.2, *p* = 0.010); 2 postoperative complications were reported in patients with OC versus 0 in those with LC Patients who received LC had a statistically significant increase in PBMC and TNF-α versus those with OC ( *p* = 0.002); a statistically significant elevation in CRP was observed in patients with LC versus OC ( *p* < 0.001) Patients with LC had a statistically significant increase in monocyte counts following surgery, compared with those with OC ( *p* = 0.001) Overall, OC-guided cholecystectomy resulted in a higher incidence of immunosuppression and surgical trauma compared with the LC approach
Chau et al 41	2002	Retrospective study	*N* = 73 [LC ( *N* = 31), OC ( *N* = 42)]	• Postoperative hospital stay duration • Morbidity • Mortality • Operative duration	This study compared the clinical outcomes of OC and LC interventions in patients (age ≥75 years) treated for acute cholecystitis	Patients with LC had a significantly reduced hospital stay duration ( *p* = 0.03) and morbidity rate ( *p* < 0.05) compared with those with OC Both study groups did not differ for major bile duct injury and mortality rate
Singh et al 42	2017	Randomized prospective study	*N* = 100 [LC ( *N* = 50), OC ( *N* = 50)]	• Operative duration • Postoperative pain duration • Hospital stay duration after surgery • Normal diet resumption following surgery • Wound infection incidence following surgery	This study compared the safety/clinical outcomes between LC and OC for cholelithiasis	Patients with LC had a comparatively lower operative duration (44.7 minutes) than those with OC (72.4 minutes) The pain duration (mean value) after surgery in patients with OC was 30.7 hours compared with 18.3 hours in those with LC The hospital stay duration (mean value) after OC was 4.8 days compared with 1.8 days after LC The normal diet restoration in LC patients was 1.2 days versus 2.1 days in those with OC Open cholecystectomy resulted in a higher incidence of postoperative wound infection than LC
Zacks et al 43	2002	Population-based cohort study	*N* = 43433 [LC ( *N* = 19662), OC ( *N* = 23771)]	• Hospital stay duration • Clinical complications/morbidity • Hospital charges • Mortality	This study utilized the NCHDAD registry to compare clinical characteristics and procedural outcomes in patients treated with LC and OC	Patients who received LC had a lower Charlson Comorbidity Index score than those with OC (4.1 vs 4.3, *p* < 0.05) The hospitalization cost for patients with LC was comparatively lower than the OC setting (USD 9139 vs USD 12125, *p* < 0.05) Patients with OC had a threefold higher risk of death compared with those with LC (95% CI: 1.4–7.3)
Agarwal et al 2023 44	2023	Single-center, prospective, observational study	*N* = 60 [LC ( *N* = 31), OC ( *N* = 29)]	• Intraoperative complications • Postoperative complications • Hernia • Umbilical port site infection • Umbilical port site hematoma • Laparoscopy to open surgery conversion requirement • Vascular injury • Visceral injury • Gas leak • Access time	This study compared the complications and outcomes of laparoscopic and open cholecystectomy	The open method required a shorter access time compared with the closed intervention. No noticeable complications or hernia were observed in either group during the study's designated follow-up duration
Khalid et al 2023 45	2023	Retrospective study	*N* = 80 [LC ( *N* = 40), OC ( *N* = 40)]	• Postoperative (Grade 1-V) complications	This study used the Clavien-Dindo classification approach to compare open and laparoscopic cholecystectomy for postoperative complications	Grade V or IV complications were not reported in either of the patient groups The incidence rates for the low-grade postoperative complications were 40% and 35% in the open procedure and laparoscopic cholecystectomy groups, respectively Except for 2.5% of those with open cholecystectomy, none of the patients in either group developed high-grade complications
Massie et al 46	1993	Retrospective study	*N* = 233 [LC ( *N* = 67), OC ( *N* = 66)]	• Morbidity/major complications	This study compared morbidity rates following LC and OC in patients treated for acute cholecystitis	Patients receiving OC had a significantly higher morbidity incidence compared with those with LC (47% vs 7%, *p* < 0.001); in addition, patients with OC had a sevenfold higher morbidity predisposition compared with LC
Wu et al 2023 47	2023	Retrospective study	*N* = 163 [LC ( *N* = 44), OC ( *N* = 119)]	• Hospital stay duration • Drain time • Blood loss • Operation time	This study compared open and laparoscopic cholecystectomy for their overall feasibility and safety	Patients who underwent laparoscopic cholecystectomy had statistically significant reductions in hospital stay duration, drain time, blood loss, and operation time, compared with those treated with open cholecystectomy (all *p* < 0.05)
Pandey and Karlatif 48	2019	Randomized prospective study	*N* = 160 [LC ( *N* = 100), OC ( *N* = 60)]	• Mean operative duration • Postoperative pain relief duration	This study investigated the benefits of LC over OC in patients treated for cholelithiasis	Patients who underwent LC had a mean operative duration of 44.7 minutes compared with 71.4 minutes for those with OC Patients with LC attained relief from the postoperative pain within 1.8 days after surgery, compared with 18.3 hours in the OC setting
Baruah et al 2022 49	2022	Randomized prospective study	*N* = 400 [LC ( *N* = 200), OC ( *N* = 200)]	• Access time • Major complications • Port-site complications • Intraoperative gas leaks	This study compared the overall efficacy and safety of open and laparoscopic cholecystectomy	The access time with open surgery was more in comparison to the closed/laparoscopic intervention (7.18 ± 2.52 versus 5.62 ± 2.23 minutes; *p* < 0.0001) Patients with laparoscopic cholecystectomy had a low incidence of intraoperative gas leaks (2/200 vs. 39/200; *p* < 0.0001) and primary port-site complications (0/200 versus 9/200; *p* = 0.0036) versus those with open cholecystectomy

Abbreviations: CBD, common bile duct; CI, confidence interval; CRP, C-reactive protein; LC, laparoscopic cholecystectomy; NCHDAD, North Carolina Discharge Abstract Database; OC, open cholecystectomy; PBMC, peripheral blood mononuclear cells; TNF-α, tumor necrosis factor-alpha.

### Mortality

**Fig. 1:**
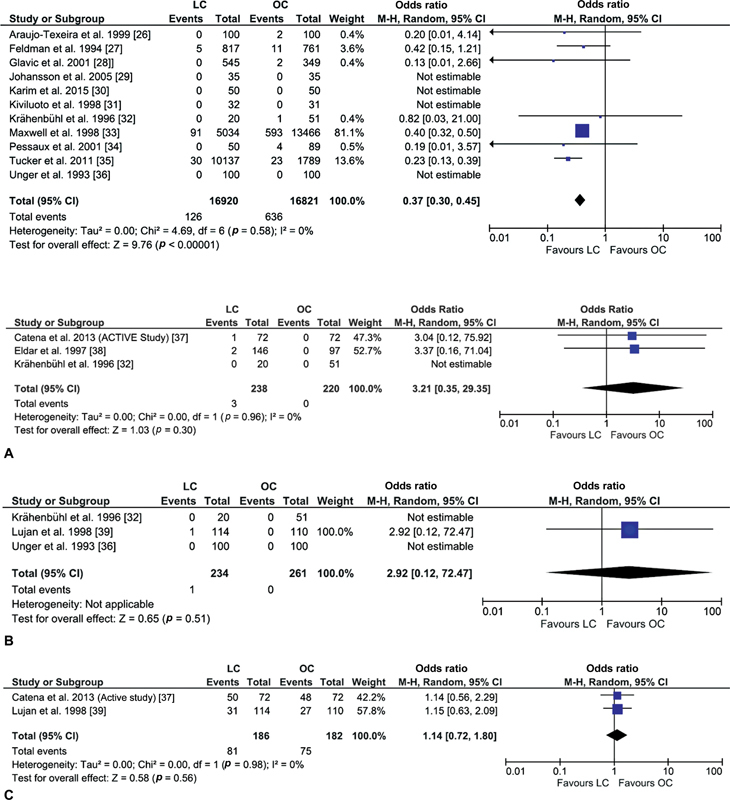
Mortality forest plot. 26 27 28 29 30 31 32 33 34 35 36 ( **A** ) Bile Leakage forest plot. 32 37 38 ( **B** ) common bile duct injury forest plot. 32 36 39 ( **C** ) Gangrene forest plot 37 39 ( **D** ) Hospital stay (days) forest plot 26 33 34 36 37 40 41 42 43 ( **E** ) Major complications forest plot 26 29 31 32 34 37 38 40 44 ( **F** ) Median hospital stay (days) forest plot. 29 31 38 45 CI, confidence interval; IV, intravenous; LC, laparoscopic cholecystectomy; OC, open cholecystectomy; SD, standard deviation.

Data from 11 studies were analyzed to compare the odds for mortality between 16,920 patients in the laparoscopic cholecystectomy group and 16,821 who underwent open cholecystectomy. 26 27 28 29 30 31 32 33 34 35 36 Patients receiving laparoscopic cholecystectomy had lower odds for mortality compared with those with open cholecystectomy (OR: 0.30, 95% CI: 0.30, 0.45, *p* < 0.00001; Fig. 1 ). A minimal heterogeneity difference was reported in the pooled outcomes; however, this finding lacked statistical significance ( *I* ^2^ = 0%, *p* = 0.58).

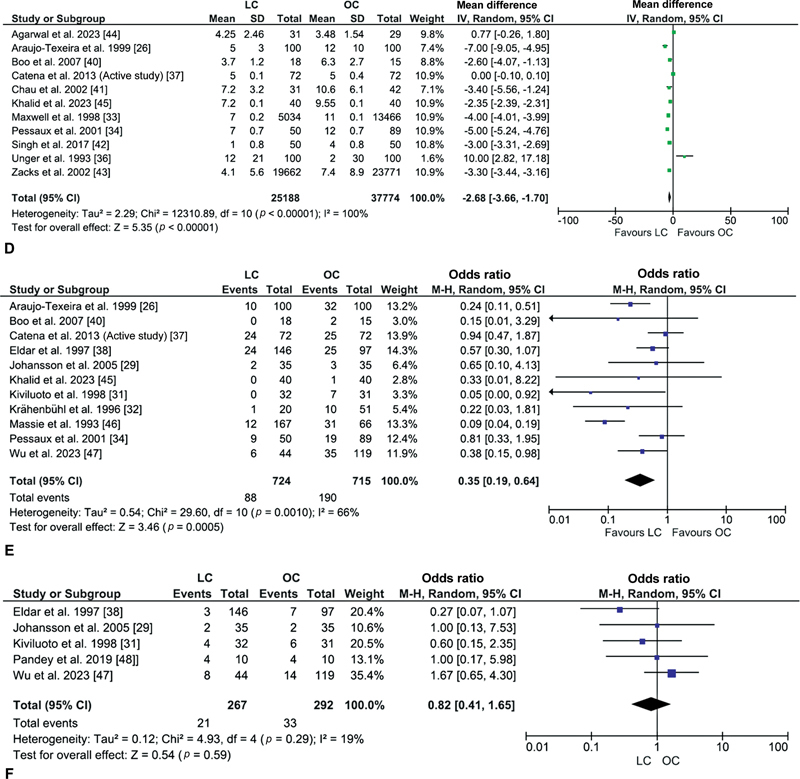



### Bile Leakage

Data from three studies were compared for bile leakage between 238 patients in the laparoscopic cholecystectomy group and 220 with open cholecystectomy. 32 37 38 Higher odds favored the possible role of open cholecystectomy in elevating the incidence of bile leakage compared with laparoscopic cholecystectomy (OR: 3.21, 95% CI: 0.35, 29.35); however, this finding lacked statistical significance ( *p* = 0.30), thereby indicating no noticeable differences for bile leakage between the study groups ( Fig. 1A ). The minimal heterogeneity difference between the included studies was also devoid of statistical significance (I ^2^ = 0%, *p* = 0.96).

### Common Bile Duct Injury

Data from three studies were compared for common bile duct injury between 234 patients receiving laparoscopic cholecystectomy and 261 with open cholecystectomy. 32 36 39 Outcomes lacked statistical significance, and common bile duct injury events were comparable between the study groups (OR: 2.92, 95% CI: 0.12, 72.47, *p* = 0.51; Fig. 1B ). A heterogeneity assessment was not possible since findings from two of the included studies were not estimable.

### Gangrene

Data from two studies were extracted to compare the odds of gangrene incidence between the study groups. 37 39 The differences in the occurrence of gangrene between 186 patients with laparoscopic cholecystectomy and 182 with open cholecystectomy were statistically insignificant (OR: 1.14, 95% CI: 0.72, 1.80, *p* = 0.56; Fig. 1C ). Similarly, low heterogeneity between the outcomes of the included studies lacked statistical significance ( *I* ^2^ = 0, *p* = 0.98).

### Mean Hospital Stays

Data from 11 studies were analyzed to compare MDs for hospital stay days between 25,188 patients in the laparoscopic cholecystectomy group and 37,774 with open cholecystectomy. 26 33 34 36 37 40 41 42 43 44 45 Pooled outcomes indicated lower means of hospital-stay days after laparoscopic cholecystectomy compared with the open procedure (MD: –2.68, 95% CI: –3.66, –1.70, *p* < 0.00001) ( Fig. 1D ). However, high heterogeneity was observed across the results of the included studies ( *I* ^2^ = 100, *p* < 0.00001).

### Major Complications

Data from 11 studies were analyzed to compare the odds of major complications between 724 patients in the laparoscopic cholecystectomy group and 715 who underwent open cholecystectomy. 26 29 31 32 34 37 38 40 45 46 47 A statistically significant outcome revealed lower odds for major complications after laparoscopic cholecystectomy compared with the open procedure (OR: 0.35, 95% CI: 0.19, 0.64, *p* = 0.0005; Fig. 1E ). The heterogeneity differences between the included studies were, however, of moderate level ( *I* ^2^ = 66%, *p* = 0.001).

### Median Hospital Stays

Data from five studies were analyzed to compare the medians of hospital stay days between 267 and 292 patients in the laparoscopic and open cholecystectomy groups, respectively. 29 31 38 47 48 Pooled results indicated no statistically significant differences in median hospital stays between the study groups (OR: 0.82, 95% CI: 0.41, 1.65, *p* = 0.59; Fig. 1F ). In addition, low heterogeneity differences between the included studies lacked statistical significance ( *I* ^2^ = 19%, *p* = 0.29).

#### Pneumonia

**Fig. 2:**
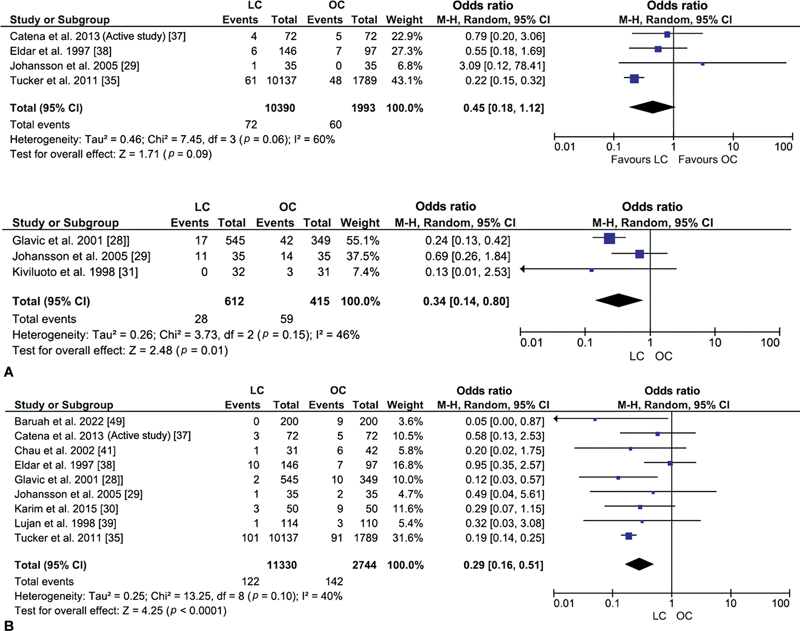
Pneumonia forest plot. 29 35 37 38 ( **A** ) Sick leaves (days) forest plot. 28 29 31 ( **B** ) Wound infection forest plot. 28 29 30 35 37 38 39 41 ( **C** ) Risk of bias in non-randomized studies of Interventions (ROBINS-I; Summary). 26 27 28 32 33 34 35 36 38 39 41 43 44 ( **D** ) ROBINS-I (Graph). ( **E** ) ROB2 (Summary). 29 30 37 31 40 42 45 ( **F** ) ROB2 (Graph). CI, confidence interval; LC, laparoscopic cholecystectomy; OC, open cholecystectomy.

Data from four studies were analyzed to compare the odds for pneumonia between 10,390 patients who underwent laparoscopic cholecystectomy and 1993 with the open procedure. 29 35 37 38 The pooled outcomes negated statistically significant differences in the incidence of pneumonia between the study groups (OR: 0.45, 95% CI: 0.18, 1.12, *p* = 0.09; Fig. 2 ). Moderate heterogeneity (I ^2^ = 60%) was observed across the outcomes of the included studies, which again lacked statistical significance ( *p* = 0.06).

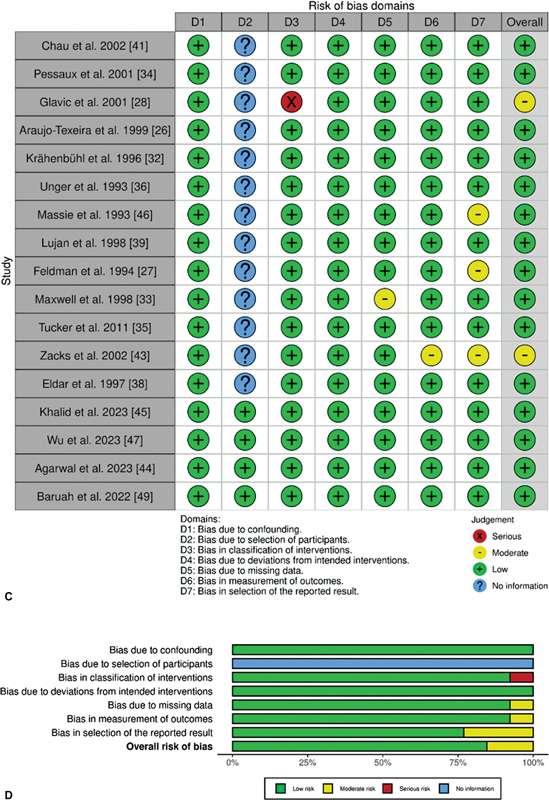

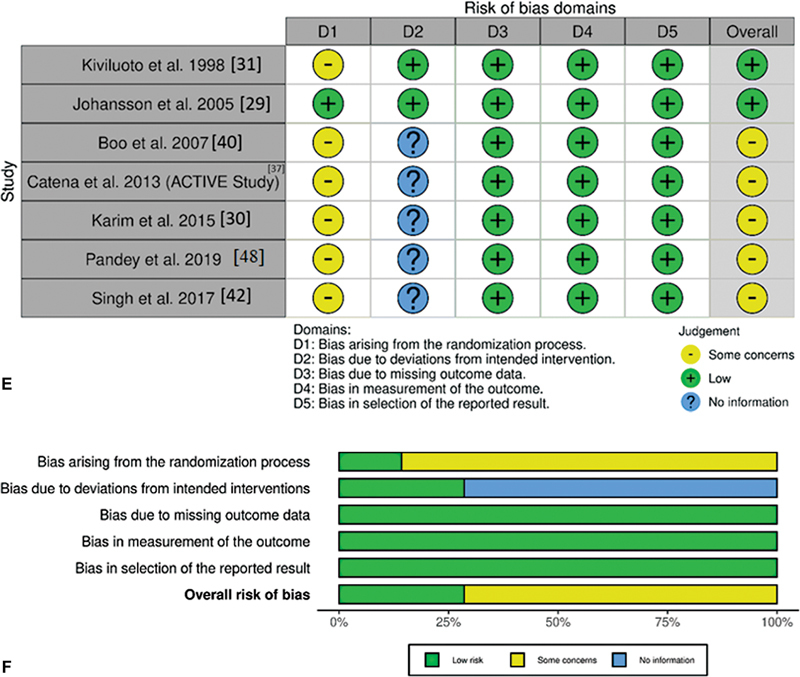





#### Sick Leaves

Data from three studies were analyzed to compare sick leaves between 612 patients in the laparoscopic cholecystectomy group and 415 who received open cholecystectomy. 28 29 31 The pooled result was statistically significant to fewer sick leaves in patients with laparoscopic cholecystectomy than those with the open procedure (OR: 0.34, 95% CI: 0.14, 0.80, *p* = 0.01 Fig. 2A ). However, the low heterogeneity differences between the included studies lacked statistical significance ( *I* ^2^ = 46%, *p* = 0.15).

#### Wound Infection

Data from nine studies were analyzed to compare wound infection events between 11,330 patients in the laparoscopic cholecystectomy group and 2744 with open cholecystectomy. 28 29 30 35 37 38 39 41 49 A statistically significant reduction in odds for wound infection was observed in patients with laparoscopic cholecystectomy compared with the open procedure (OR: 0.29, 95% CI: 0.16, 0.51, *p* < 0.0001; Fig. 2B ). In addition, outcomes from the included studies had minimal heterogeneity differences that lacked statistical significance ( *I* ^2^ = 40%, *p* = 0.1).

#### ROB Outcomes

The overall outcomes from the ROBINS-I measure indicated a low ROB in 11/13 and a moderate ROB in 2/13 of nonrandomized retrospective/prospective studies ( Fig. 2C ). None of the nonrandomized studies provided any information on bias based on the inclusion of subjects. Only 1/13 nonrandomized studies reported a serious ROB concerning the intervention classification. Five studies were associated with ROB due to outcome measurement/missing data/result selection ( Fig. 2D ). The overall statistical assessment of randomized trials indicated some ROB concerns in 4/7 studies, and low ROB in 2/7 studies ( Fig. 2E ). Five studies provided no information about possible bias due to intervention deviation. In addition, low ROB was reported for missing outcome data, outcome measurement, and outcome reporting ( Fig. 2F ). Some concerns for ROB were reported for the randomized process in 6/7 studies.

## Discussion

The pooled outcomes from this meta-analysis revealed a statistically significant reduction in mortality, mean hospital stay duration, major complications, and sick leaves in patients who underwent laparoscopic cholecystectomy compared with those with the open intervention. In addition, no statistically significant differences were recorded for bile leakage, common bile duct injury, gangrene, median hospital stay days, and pneumonia between the study groups. These findings indicate the higher impact of laparoscopic cholecystectomy in improving patient outcomes, including safety episodes, compared with open cholecystectomy.

Findings from this study concord with the meta-analysis by Coccolini et al emphasizing statistically significant reductions in the mean hospital stay duration in patients with laparoscopic cholecystectomy versus the open procedure. 14 Additionally, the results strengthened the outcomes of Coccolini et al that indicated a decline in postoperative morbidity, by affirming reductions in major postoperative complications in the laparoscopic cholecystectomy group compared with those with open cholecystectomy. 14 The findings of this study also favored the meta-analysis outcomes of Antoniou et al that indicated lower morbidity and mortality in patients with laparoscopic cholecystectomy compared with the open intervention. 13 They further strengthened the meta-analysis findings of de Goede et al indicating lower hospital stay duration and minimal postoperative complications after laparoscopic cholecystectomy compared with the open procedure. 50

The latest evidence reveals a marked reduction in interleukin-8 (IL-8), IL-6, and C-reactive protein levels in patients with laparoscopic cholecystectomy. 51 In addition, rapid recovery after laparoscopic cholecystectomy is due to its noninvasive approach and the attainment of a wider surgical field via the endoscopic device. A reduced level of inflammatory response and minimal trauma are the other significant factors attributing to the success of laparoscopic cholecystectomy in patients with gallbladder disease. 51 Although our study did not delineate any significant differences in bile duct injury/leakage between open- and laparoscopic cholecystectomies, literature reviews indicate the lower risk of these events following the laparoscopic approach due to minimal surgical incision. Since the laparoscopic technique minimally interferes with organ function, patients have a lower risk of intraabdominal bleeding and bile duct injury. Evidence also indicates the attainment of elevated quality of life after laparoscopic cholecystectomy due to a significant decline in clinical complication rates, compared with the open technique. 51 Lastly, a 98% cure rate after laparoscopic cholecystectomy is due to higher postoperative recovery, low body impact, and limited operative trauma.

## Limitations

Like any other meta-analysis, this study is not devoid of noticeable limitations. First, due to the data availability limitation, the authors could not perform a subgroup analysis for assessing cardiovascular outcomes in patients with open cholecystectomy and those treated with a laparoscopic procedure. Second, the evaluation of laparoscopic-to-open cholecystectomy conversion rates was not possible due to inconsistencies in the reported data. Third, findings from this study hold limited value for pediatric patients since most of the included studies analyzed cholecystectomy outcomes in adult/elderly patients. Fourth, the authors could not compare outcomes of both procedures by meta-regression, based on comorbidities, symptoms, body mass index, and past treatments due to data limitations. Fifth, the authors could not perform a subgroup analysis or meta-regression assessment of the study end-points for gallbladder disease manifestations, due to data scarcity. Finally, the inclusion of a highly heterogeneous group of studies for quantitative analysis impacted the overall generalizability of outcomes.

## Conclusion

The findings from this systematic review and meta-analysis favor the role of laparoscopic cholecystectomy over open cholecystectomy in minimizing the incidence of mortality, mean hospital stay duration, major complications, and sick leaves. Furthermore, comparable outcomes including bile leakage, common bile duct injury, gangrene, median hospital stay days, and pneumonia events between both study groups reaffirm the need to replace the open procedure with laparoscopic cholecystectomy for patients with gallbladder disease.
