# Supplementary material for: A Systematic Review and Meta-Analysis of the Outcomes of Laparoscopic Cholecystectomy Compared to the Open Procedure in Patients with Gallbladder Disease
Source: Avicenna J Med. 2024 Feb 1;14(1):3–21. doi: 10.1055/s-0043-1777710 (PMC11057899; doi:10.1055/s-0043-1777710)
Supplement: Supplementary file 1 — Supplementary Material [file 10-1055-s-0043-1777710-s230053.pdf]

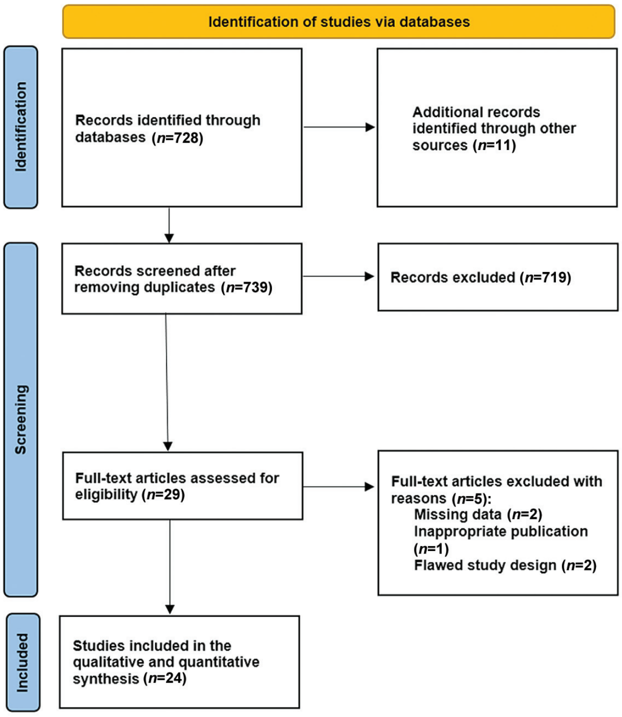

Supplementary Fig. S1 Preferred reporting items for systematic reviews and meta-analyses (PRISMA) flow diagram.

 © 2024. The Author(s).
